# Nasoethmoid Schwannoma with Intracranial Extension: A Case Report and Comprehensive Review of the Literature

**DOI:** 10.7759/cureus.3182

**Published:** 2018-08-22

**Authors:** Daniel G Eichberg, Simon A Menaker, Simon S Buttrick, Sakir H Gultekin, Ricardo J Komotar

**Affiliations:** 1 Neurological Surgery, University of Miami Miller School of Medicine, Miami, USA; 2 Neurological Surgery, University of Miami Miller School of Medicine , Miami, USA; 3 Pathology, University of Miami Miller School of Medicine, Miami, USA

**Keywords:** schwannoma, nasoethmoid, anterior fossa, brain tumor, skull base, neurosurgery, otolaryngology

## Abstract

We describe all cases of nasoethmoid schwannomas with intracranial extension reported in the literature, including an original case report describing the successful gross total resection of a nasoethmoid schwannoma with intracranial extension. Ten cases of nasoethmoid schwannoma with intracranial extension have previously been reported. These lesions most often appear in the second through fourth decades of life and commonly present with anosmia, headache, and visual deficits. Bifrontal craniotomy was the predominantly implemented surgical approach and gross total resection was achieved in all cases, except for one. In conclusion, nasoethmoid schwannoma with intracranial extension is a rare disease entity that is most often benign and is most commonly treated by gross total resection using a bifrontal craniotomy approach.

## Introduction

Nasoethmoid schwannomas are uncommon tumors of the nasal cavity and paranasal sinuses that arise from local peripheral nerve sheaths. Although schwannomas frequently occur in the head and neck, fewer than 50 cases in the nasoethmoid region have been reported in the medical literature [1]. Of these 50 reported cases, only a small minority extend intracranially to involve the orbit or anterior cranial fossa. The vast majority of these lesions are benign, encapsulated, and spread locally by eroding the bony structures in which they reside [2], although a handful of malignant schwannomas involving the paranasal sinuses have been reported [3]. These tumors often display few early symptoms (in one case, none at all [4]) and develop within complex anatomic structures, which results in their characteristically late detection [5].

Herein, we present the case of a nasoethmoid schwannoma with intracranial extension. We discuss the pertinent clinical, operative, and histopathological findings. We also performed a systematic review of the literature of all reported cases of nasoethmoid schwannoma with intracranial extension.

The PubMed database and all major neurosurgery journals were searched during March of 2018 using the keywords “nasoethmoid”, “naso-ethmoid”, “schwannoma”, and “intracranial extension”, alone or in combination to obtain articles fitting the inclusion and exclusion criteria. The inclusion criteria were schwannomas involving the nasal cavity or paranasal sinuses with intracranial extension. Schwannomas without intracranial extension were excluded.

## Case presentation

**Figure 1:**
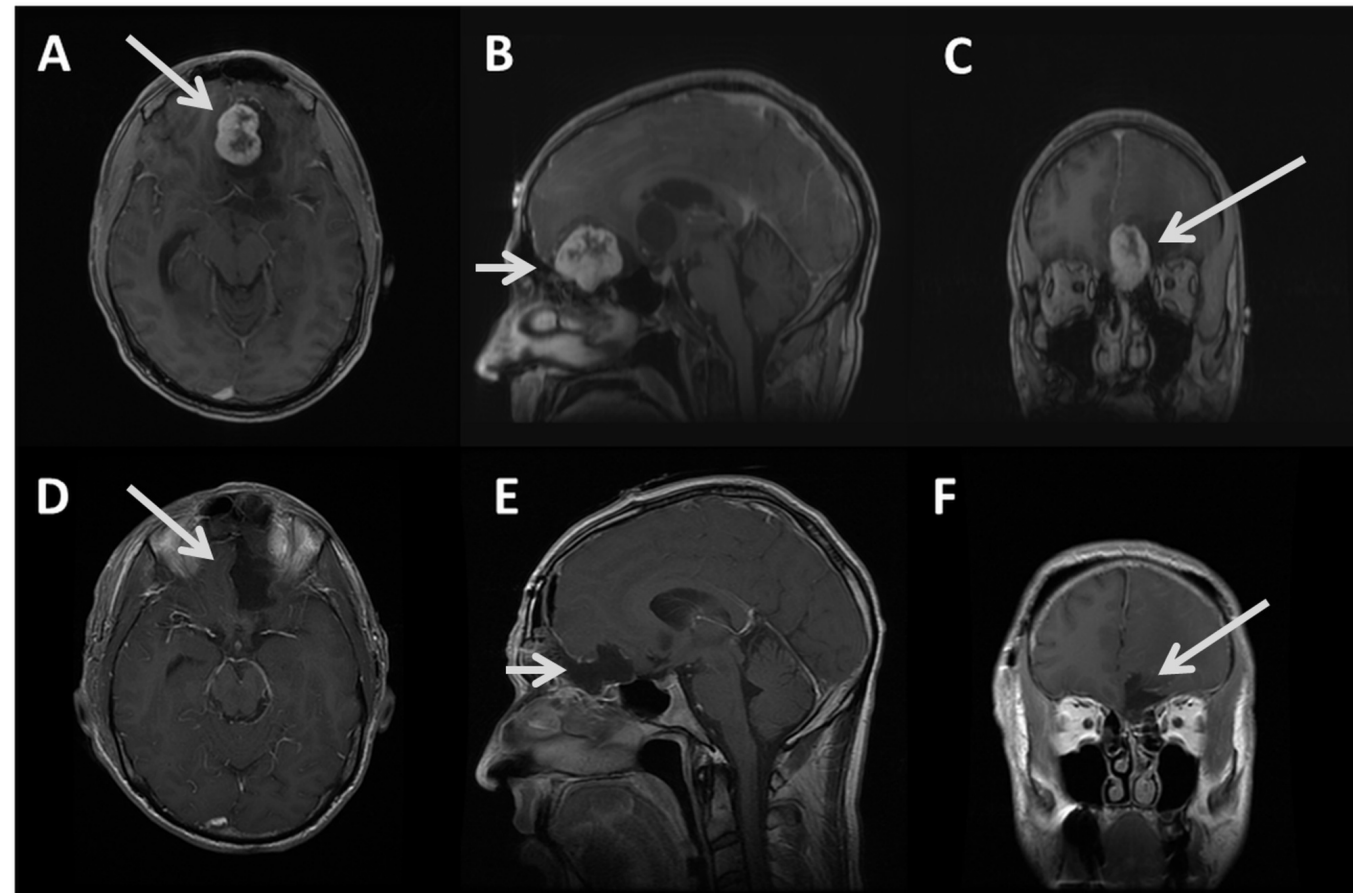
Pre- and postoperative brain magnetic resonance imaging (MRI) demonstrating gross total resection of nasoethmoid schwannoma with intracranial extension Preoperative axial (A) sagittal (B) and coronal (C) gadolinium enhanced MRI demonstrating cystic and heterogeneously enhancing solid mass (arrows) extending from the mid-left ethmoid air cells into the anterior cranial fossa, left frontal lobe, and left basal ganglia, causing mass effect on the left lateral ventricle and midline shift. Postoperative axial (C) sagittal (D) and coronal (E) gadolinium enhanced MRI demonstrating gross total resection (arrows demonstrating resection cavity with no residual tumor).

A 41-year-old male was referred to our department for possible neurosurgical intervention following magnetic resonance imaging (MRI) that showed a lesion suspected to be an esthesioneuroblastoma (Figure 1). He presented with a several-month history of intermittent headaches and loss of smell. He also reported near fainting episodes and slight unintentional weight loss over the same time period. The review of symptoms was negative for epistaxis and visual deficits, and neurological examination showed no abnormalities other than his aforementioned anosmia.

His MRI showed a large mass with both cystic and heterogeneously enhancing solid components extending from the region of the mid-left ethmoid air cells superiorly into the anterior cranial fossa, left frontal lobe, and left basal ganglia, causing mass effect on the left lateral ventricle and a midline shift.

The tumor was resected through a bifrontal craniotomy; the original surgical plan involved a formal craniofacial resection via a combined neurosurgical-otolaryngological approach. Intraoperatively, the tumor was found to have a clear arachnoid plane with no gross attachment to the surrounding dura or brain, and frozen section did not reveal any atypia, increased mitoses, or other aggressive features. Therefore, we elected to forgo a full craniofacial resection. Gross total resection was achieved and the skull-based repair was completed with a harvested pericranial flap and watertight dural closure. The patient awoke at his neurologic baseline and was discharged home on postoperative day one.

**Figure 2:**
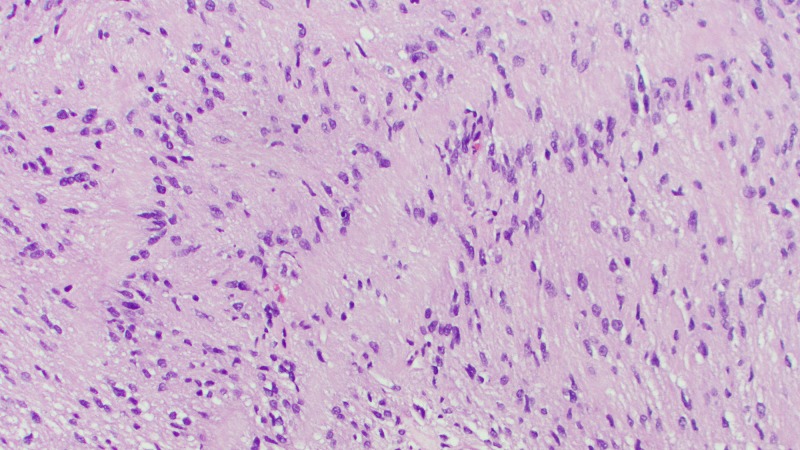
Surgical pathology slide from the current case report demonstrating Verocay bodies under x200 power

**Figure 3:**
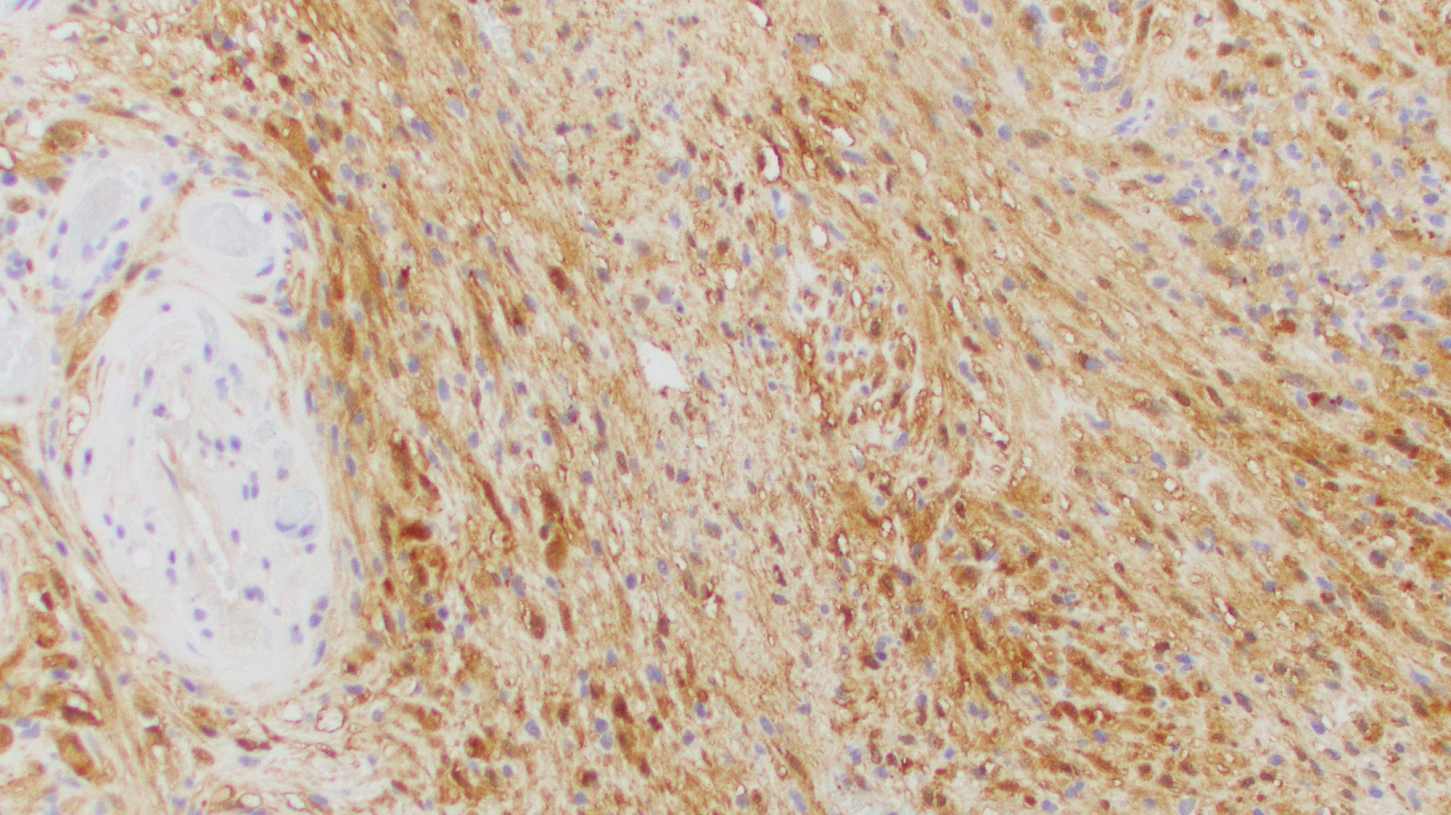
Surgical pathology slide from the current case report demonstrating labeling of schwannoma cells with S-100 protein immunohistochemical stain

**Figure 4:**
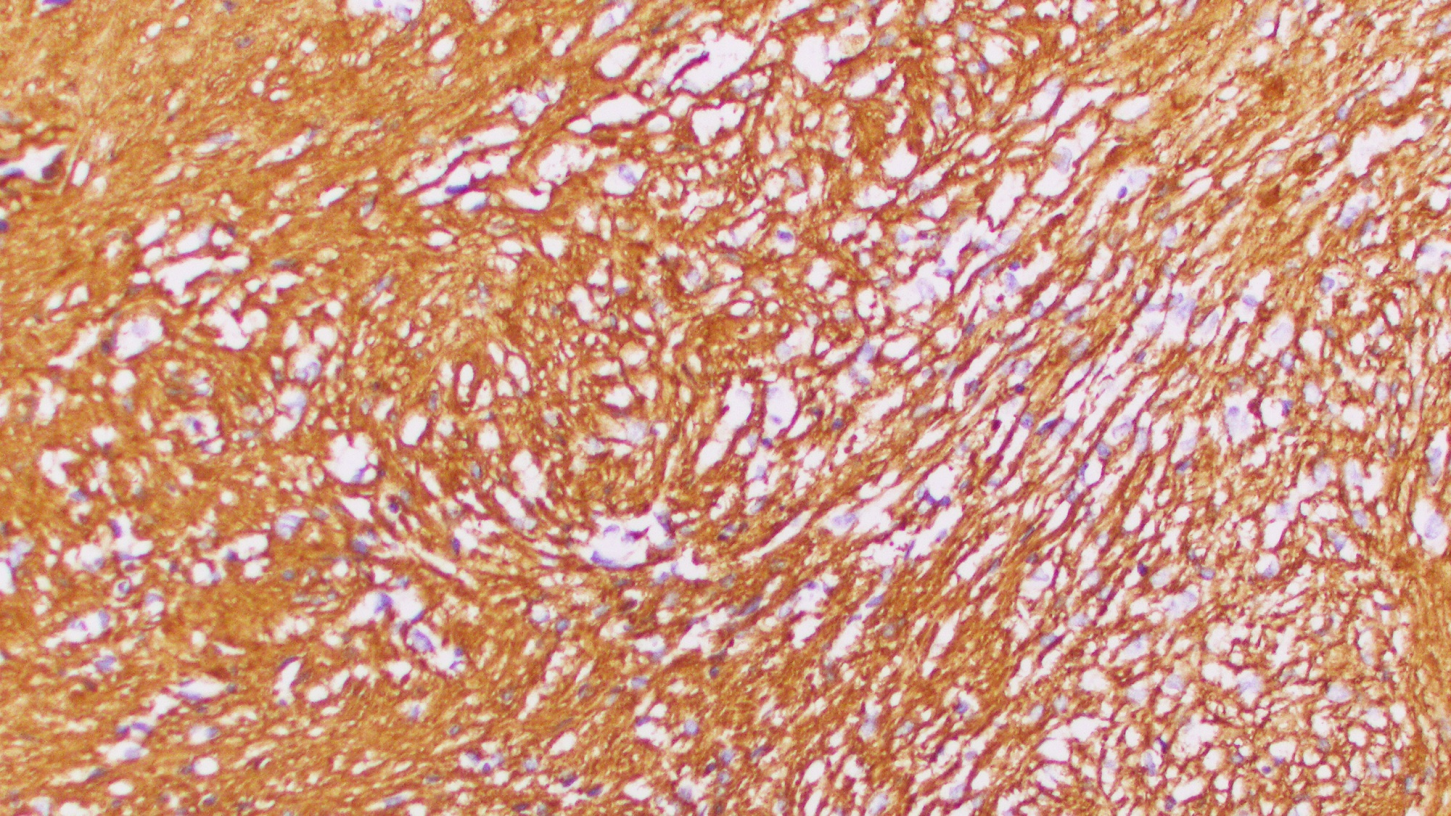
Surgical pathology slide from the current case report demonstrating labeling of schwannoma cells with collagen type IV immunohistochemistry, demonstrating basal lamina in individual cells

**Figure 5:**
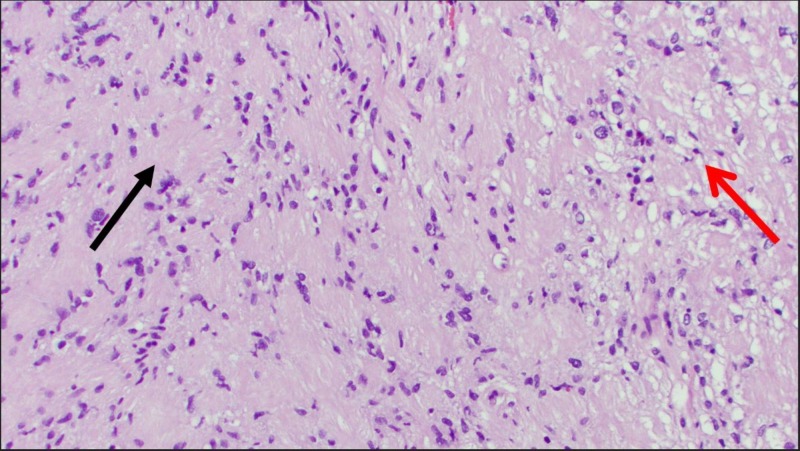
Surgical pathology slide from the current case report demonstrating schwannoma on hematoxylin and eosin (H&E) stain with x200 power. Antoni A (black arrow on left) and Antoni B (red arrow on right) areas are demonstrated.

On permanent section, the lesion was determined to be a World Health Organization (WHO) Grade I schwannoma (Figures 2-5). Surgical pathology demonstrated S-100 protein immunohistochemical stain positivity and was negative for meningioma markers (progesterone receptor (PR) and anti-epithelial membrane antigen (EMA)). The patient had an uneventful postoperative course and experienced no significant headaches, neurologic symptoms, cerebrospinal fluid (CSF) rhinorrhea, or other notable complications.

Literature review

We identified 10 cases of nasoethmoid schwannoma with intracranial extension in the medical literature (Table 1). These lesions occurred in patients from age 24 to 50, with the vast majority presenting by age 40. There appears to be no association with gender. Although specific symptoms depend on the location and size of each tumor, most patients displayed a gradual onset of anosmia with increasing tumor growth and extension. Headache and visual deficits were also commonly seen among symptomatic patients. Bifrontal craniotomy was the most frequently utilized surgical approach for tumor resection, although several cases required a combined intracranial and transfacial approach. Gross total resection was achieved in all cases except one; the exception was a malignant tumor, which was neither encapsulated nor easily separable from the surrounding anatomical structures like the other benign tumors. Among articles reporting postoperative complications, CSF rhinorrhea was seen in three patients, as well as a variety of other complications associated with the unique locations and operative courses of individual lesions (Table 1). Two patients, including the case presented in this paper, had no postoperative complications.

**Table 1:** Summary of literature on nasoethmoid schwannomas with intracranial extension Y: yes; N: no; M: male; F: female.

Author	Year	Patient Age	Patient Gender	Presenting Symptoms	Surgical Approach	Gross Total Resection?	Postoperative Complications	Follow-up Length
George et al. [1]	2009	27	F	Blurred vision; headache	Frontal craniotomy	Y	CSF rhinorrhea	Not specified
Bavetta et al. [2]	1993	41	M	Anosmia; blurred vision; nasal obstruction	Frontal craniotomy; medial maxillary osteotomy	Y	Enophthalmos; hematoma	Not specified
Ogunleye et al. [3]	2006	31	F	Anosmia; mid-facial swelling; unilateral blindness	Not specified	N	Adjuvant radiotherapy	Not specified
Hong et al. [4]	2016	24	M	Asymptomatic	Bifrontal craniotomy	Y	Abscess; CSF rhinorrhea; displacement of intranasal fat graft; headache	Not specified
Sharma et al. [5]	1998	35	M	Anosmia; epistaxis; nasal obstruction; seizures	Bifrontal craniotomy	Y	CSF rhinorrhea	6 months
Zovickian et al. [6]	1986	40	M	Headache; nasal congestion	Frontal craniotomy; intranasal	Y	Not specified	Not specified
Enion et al. [7]	1991	28	M	Headache	Bifrontal craniotomy	Y	CSF rhinorrhea	3 months
Fujiyoshi et al. [8]	1997	38	M	Epistaxis; nasal congestion	Not specified	Y	Not specified	Not specified
Gatscher et al. [9]	1998	50	F	Anosmia; headache; visual deterioration	Bifrontal craniotomy	Y	Not specified	Not specified
Siqueira et al. [10]	2001	40	F	Anosmia; frontal deformity; headache	Bifrontal craniotomy; lateral rhinotomy	Y	None	5 years
Eichberg et al. [11]	2017	41	M	Anosmia; headache	Bifrontal craniotomy	Y	None	1 year

## Discussion

Schwannomas of the nasoethmoid region are rare tumors, the majority of which are benign, grow slowly, and evade early detection. The extension of these lesions into the intracranial compartment is a particularly uncommon occurrence, as this is only the eleventh reported case in the literature (Table 1). Nasoethmoid schwannoma spread is facilitated by the gradual destruction of overlying bone, especially the cribriform plate, orbital wall, and roof of the ethmoid air cells. While there are many peripheral nerve branches that innervate the nasal cavity and paranasal sinuses, nasoethmoid schwannomas most likely arise from regular Schwann cells encasing either sensory branches of the ophthalmic and maxillary divisions of the trigeminal nerve or local autonomic nerves [6-7].

There are no specific radiological findings on MRI or computed tomography (CT) that differentiate schwannomas from other contrast enhancing lesions of the nasal cavity and anterior cranial fossa. The differential diagnosis should include esthesioneuroblastoma, papilloma, meningioma, nasopharyngeal carcinoma, sarcoma, and lymphoma [1]. The lack of pathognomonic radiographic characteristics necessitates histopathological examination for accurate diagnosis of nasoethmoid schwannomas.

Our comprehensive review of the literature highlights several important patterns and characteristics regarding the presentation and treatment of these tumors. First, gross total resection was achieved for all benign tumors. Only one of the articles detailed a malignant schwannoma, which was treated with subtotal resection followed by adjuvant radiation with no resultant complications. As gross total resection was achieved in 91% of cases, this demonstrates that nasoethmoid schwannomas with intracranial extension are highly amenable to resection, despite their occasionally impressive size.

Bifrontal craniotomy was the preferred surgical approach. In most cases, the intranasal portion of the tumor could be removed superiorly through the skull base defect, with the widening of the defect, if necessary, which represents a less invasive option than the traditional intracranial-transfacial approach for tumors with both intracranial and extracranial components [5]. The skull based defect can typically be repaired with a pericranial flap, as was done in our case.

At our institution, we prefer bifrontal craniotomy for midline anterior cranial fossa lesions such as nasoethmoid schwannomas because of the expansive tumor exposure and wide operative working angles this approach provides. During closure, after primary repair of the dura, we overlay a sheet of dehydrated amniotic membrane over the dura to minimize the risk of CSF leak [11]. However, for smaller tumors, or tumors in which the intracranial tumor extension is less than 50%, we would consider endoscopic endonasal approaches to these tumors. The advantages of endoscopic endonasal approaches include less brain retraction, more direct tumor visualization, and less cosmetically disfiguring incision [12].

It is important to note that over half of all patients presented with one or more nasal symptoms including anosmia, epistaxis, congestion, and overt obstruction. Accordingly, nasoethmoid schwannoma with intracranial extension should be considered in the setting of masses involving the nasal cavity, paranasal sinuses, or anterior skull base that present with persistent nasal symptoms. However, we caution against ruling out this type of lesion in the absence of nasal symptoms because several patients with predominantly intracranial tumors presented with no such complaints [1,4].

Finally, because CSF leak occurred in 36% of cases, particular attention should be directed towards the skull base reconstruction upon resection of a nasoethmoid schwannoma with intracranial extension due to its involvement of both the intra- and extracranial compartments. We attribute our prevention of postoperative CSF leaks in part to the increasing use of dehydrated amniotic membrane products [11].

## Conclusions

Nasoethmoid schwannoma with intracranial extension is a rare condition which can mimic several other, more aggressive tumors on imaging. In most cases, they can be completely resected through a bifrontal craniotomy. We report the eleventh case of nasoethmoid schwannoma with intracranial extension in the medical literature and provide a systematic review of all previously reported cases.

## References

[1] George KJ, Price R (2009). Nasoethmoid schwannoma with intracranial extension. Case report and review of literature. Br J Neurosurg.

[2] Bavetta S, McFall MR, Afshar F, Hutchinson I (1993). Schwannoma of the anterior cranial fossa and paranasal sinuses. Br J Neurosurg.

[3] Ogunleye AO, Ijaduola GT, Malomo AO (2006). Malignant schwannoma of the nasal cavity and paranasal sinuses in a Nigerian. Afr J Med Med Sci.

[4] Hong CS, Pomeraniec IJ, Starke RM, Shaffrey ME Nasoethmoid schwannoma with intracranial extension. Case report and review of the literature. Br J Neurosurg.

[5] Sharma R, Tyagi I, Banerjee D, Pandey R (1998). Nasoethmoid schwannoma with intracranial extension. Case report and review of literature. Neurosurg Rev.

[6] Zovickian J, Barba D, Alksne JF (1986). Intranasal schwannoma with extension into the intracranial compartment: case report. Neurosurgery.

[7] Enion DS, Jenkins A, Miles JB, Diengdoh JV (1991). Intracranial extension of a naso-ethmoid schwannoma. J Laryngol Otol.

[8] Fujiyoshi F, Kajiya Y, Nakajo M (1997). CT and MR imaging of nasoethmoid schwannoma with intracranial extension. AJR Am J Roentgenol.

[9] Gatscher S, Love S, Coakham HB (1998). Giant nasal schwannoma with intracranial extension. Case illustration. J Neurosurg.

[10] Siqueira MG, Jennings E, Moraes OJ (2001). Naso-ethmoid schwannoma with intracranial extension: case report. Arq Neuropsiquiatr.

[11] Eichberg DG, Ali SC, Buttrick SS, Komotar RJ (2018). The use of dehydrated amniotic membrane allograft for the augmentation of dural repair in craniotomies. Cureus.

[12] Blake DM, Husain Q, Kanumuri VV, Svider PF, Eloy JA, Liu JK (2014). Endoscopic endonasal resection of sinonasal and anterior skull base schwannomas. J Clin Neurosci.

